# Antidepressants and suicidal behaviour in late life: a prospective population-based study of use patterns in new users aged 75 and above

**DOI:** 10.1007/s00228-017-2360-x

**Published:** 2017-11-04

**Authors:** Khedidja Hedna, Karolina Andersson Sundell, Armina Hamidi, Ingmar Skoog, Sara Gustavsson, Margda Waern

**Affiliations:** 1 Department of Psychiatry and Neurochemistry, Institute of Neuroscience and Physiology, Sahlgrenska Academy, University of Gothenburg, SE-413 45 Gothenburg, Sweden; 2 Section of Epidemiology and Social Medicine, Department of Public Health and Community Medicine at Institute of Medicine, University of Gothenburg, Gothenburg, Sweden; 3 Medical Evidence and Observational Research, AstraZeneca, Mölndal, Sweden; 4 Health Metrics, The Sahlgrenska Academy, University of Gothenburg, Gothenburg, Sweden

**Keywords:** Antidepressants, Suicide, Pharmacoepidemiology, Aged, Cohort study

## Abstract

**Purpose:**

To investigate associations between antidepressant use patterns and risk of fatal and non-fatal suicidal behaviours in older adults who initiated antidepressant therapy.

**Method:**

A national population-based cohort study conducted among Swedish residents aged ≥ 75 years who initiated antidepressant treatment. Patients who filled antidepressant prescriptions between January 1, 2007 and December 31, 2013 (*N* = 185,225) were followed until December 31, 2014. Sub-hazard ratios of suicides and suicide attempts associated with use patterns of antidepressants, adjusting for potential confounders such as serious depression were calculated using the Fine and Gray regression models.

**Results:**

During follow-up, 295 suicides and 654 suicide attempts occurred. Adjusted sub-hazard ratios (aSHRs) were increased for both outcomes in those who switched to another antidepressant (aSHR for suicide 2.42, 95% confidence interval 1.65 to 3.55, and for attempt 1.76, 1.32 to 2.34). Elevated suicide risks were also observed in those who concomitantly filled anxiolytics (1.54, 1.20 to 1.96) and hypnotics (2.20, 1.69 to 2.85). Similar patterns were observed for the outcome suicide attempt. Decreased risk of attempt was observed among those with concomitant use of anti-dementia drugs (0.40, 0.27 to 0.59).

**Conclusion:**

Switching antidepressants, as well as concomitant use of anxiolytics or hypnotics, may constitute markers of increased risk of suicidal behaviours in those who initiate antidepressant treatment in very late life. Future research should consider indication biases and the clinical characteristics of patients initiating antidepressant therapy.

**Electronic supplementary material:**

The online version of this article (10.1007/s00228-017-2360-x) contains supplementary material, which is available to authorized users.

## Background

Older populations, in particular older men, tend to have the highest rates of suicide in many countries worldwide [1, 2]. Depression is common in older adults who die by suicide, and it has been estimated that three quarters of all late-life suicides could be prevented, if depression could be successfully treated [3].

There is some evidence that antidepressant therapy is associated with decreased risk of suicide in late life [4, 5]. However, there is a need for studies that focus specifically on the oldest segment of the population. Clinical trials tend to exclude older persons often due to physical frailty [6]. This is problematic as medication use patterns, treatment response and side effects may all differ between younger and older users of antidepressants, due to higher levels of psychiatric and somatic comorbidities in the older group, as well as age-related physiological changes [7, 8].

Given the fact that the identification and subsequent treatment of late life depression is a major suicide prevention strategy [9, 10], it is surprising that only a few pharmacoepidemiological studies investigate how different use patterns of antidepressants may impact on the risk of suicide in older adults [11, 12]. We could find no population-based studies providing risk estimates for concomitant use of psychoactive drugs in this high-risk age group. The latter is important as sedatives and hypnotics are widely prescribed for older patients with depression, and these medications may be related to increased risk of suicide [13, 14]. We have previously presented data demonstrating that anxiolytics and hypnotics, but not antidepressants, were associated with increased suicide risk in older adults after adjustment for confounding by indication [15]. That study was small and based on retrospective information, and potential associations with suicidal behaviour need to be examined using large, prospective data sets.

The aim of the present study was to investigate the association between antidepressant use patterns and the risk of suicide and suicide attempt in a national population-based cohort of older Swedish adults who initiated antidepressant therapy. Considering the fact that suicide rates are particularly high in the oldest age group, we chose to focus on those aged 75 years and above.

## Methods

### Study design

We conducted a prospective cohort study employing data from several national registers, linked by the unique personal identity number [16].

### Study population and study period

The study population comprised all Swedish residents aged ≥ 75 years who initiated antidepressant treatment between January 1, 2007 and December 31, 2013. Those who filled a prescription for an antidepressant during this period with no such fills during the preceding year were considered treatment initiators. The new-user design minimised confounding [17]. The cohort (*N* = 185,225) was followed from the date of first filled prescription, denoted the index date, until December 31, 2014 or until migration or death. The study flow diagram is presented in Online Resource 1.

### Data sources

The Swedish Prescribed Drug Register (SPDR) was used to identify antidepressant initiators and patterns of drug use. The SPDR has full coverage of filled prescriptions in outpatient care [18]. It includes information on dispensed medications, dispensed quantity, number of defined daily doses and prescribed daily doses in free text. The National Patient Register [19] was used to identify individuals with diagnosis of suicide attempt. This register includes up to 30 diagnoses for specialised in- and outpatient care based on the International Classification of Diseases, 10th version (ICD-10). Thus, the National Patient Register captures cases of depression requiring specialist services. For the purpose of this study, individuals with a depression diagnosis, as identified by the National Patient Register, are considered to have serious depression. Data on cause of death, including suicide, were collected from the Cause of Death Register. Persons residing in nursing homes were identified by social service data from the National Board of Health and Welfare. Statistics Sweden provided information on migration during the follow-up period.

### Exposure measures: medication use patterns

Antidepressants were defined as substances included in the Anatomical Therapeutic Chemical group (ATC-group) N06A [20]. We grouped antidepressants into major classes: tricyclic antidepressants (TCAs) (N06AA), selective serotonin reuptake inhibitors (SSRIs) (N06AB), serotonin-norepinephrine reuptake inhibitors and norepinephrine reuptake inhibitors (SNRIs/NRIs) (N06AX21, N06AX16, N06AX18), and other antidepressant medications.

We defined early discontinuation as not refilling any antidepressant medication within 180 days after the index date. Combination use was defined as the fill of two or more different antidepressants within 180 days following the index date, with the first antidepressant refilled after the purchase of the second antidepressant. Switching was defined as filling two or more different antidepressant substances within 180 days following the index date without a new refill of the index antidepressant after the fill of the new antidepressant. In Sweden, patients can purchase 3 months’ supply at each fill within the Pharmaceutical Benefits Scheme; in practice, packages of approximately 100 units are often dispensed. In addition, start packages covering up to 1 month’s supply of medication are sometimes dispensed when initiating a new treatment. To avoid misclassifying new users who did not have a start package when initiating their therapy, or with late refill of their medications, we applied the conservative period of 180 days after initiating the therapy to define different use patterns of antidepressants.

We estimated the patients’ adherence to their antidepressant therapy by the medication possession ratio (MPR), which measures the proportion of days’ supply during the observation period. The number of days’ supply was calculated by dividing the total number of filled prescribed doses by the number of estimated prescribed daily doses (PDDs). We estimated a theoretical substance-specific PDD based on a review of drug strength and dosage instructions among a sample of the study population, and a PDD/defined daily dose (DDD) was calculated for each substance. We assumed that the theoretical PDD was similar for multidose users [21]. For substances with few users and for those lacking dosage instructions, a theoretical PDD was set at 0.5 DDD. Non-adherence was defined as MPR < 80% [22].

Concurrent use of other psychotropic medications was defined as filling a prescription for another psychotropic within the 90 days following the refill of an antidepressant. Psychotropic medications were classified as follows: antipsychotics (N05A), anxiolytics (N05B), sedatives (N05C), mood stabilisers (N03AF01, N03AG01, N03AX09, N05AN01), and anti-dementia drugs (N06D).

### Outcome measures

The study outcomes were identified using the following ICD-10 codes: intentional self-harm (X60-X84), harm of undetermined intent (Y10-Y34), and sequelae of intentional self-harm and of events of undetermined intent (Y87.0 and Y87.2) from the Cause of Death Register (suicide) and from the National Patient Register (attempted suicide).

### Covariates

Potential confounders included age at index date, sex, suicide attempt during the year preceding the index date, index antidepressant medication, and serious depression in the 3 months preceding or following the index date. Use of statins was used as a proxy of cardiovascular comorbidity since previous studies have shown associations between cardiovascular comorbidity, including cerebrovascular disease, and both depression [23] and suicide [24]. Nursing home residence during the year preceding the index date was used as a general marker of frailty, which may be associated with both depression [25] and suicide [26].

### Statistical analysis

We used the Fine and Gray proportional hazard models [27] to estimate the sub-hazard ratios (SHRs) with 95% confidence intervals for the association between use patterns and suicide or suicide attempts. The Fine and Gray model is a time-to-event model, similar to the Cox proportional hazard model. However, unlike Cox’s regression, the Fine and Gray model is developed for competing data and takes into account that participants may die of other causes than that of interest. Use patterns were included in the model as time-varying covariates and the models were adjusted for the potential confounders mentioned above. In addition, we conducted gender-stratified analyses. Due to partially missing data, 210 individuals were excluded from the regression analyses.

We also described the crude associations between the use patterns of antidepressants and baseline characteristics including index medication (Online Resource 2). In order to assess the robustness of our findings, we used the Cox proportional hazards models to estimate the hazard ratios for cause-specific risk. A 0.05 significance level was used. Data management and statistical analyses were performed using SAS version 9.4 (SAS Institute, Cary, NC).

The study was approved by the Regional Ethical Review Board in Gothenburg (no: 111-15) as well as security clearance by the register holders in accordance with national regulations.

## Results

Table 1 describes the baseline characteristics of the study population. Almost two thirds of the new antidepressant users were women. The mean age was 83.4 years, and almost one fifth lived in nursing homes. Four out of one thousand new users had attempted suicide during the year preceding the initiation of the antidepressant therapy, and 4% had a serious depression according to the National Patient Register. Selective serotonin reuptake inhibitors were filled by almost two thirds of the new users. Concomitant treatment with at least one other psychotropic medication was present for over half of the study population. The median length of follow-up was 2.6 (interquartile range 1.2–4.6) years.

**Table 1:** Baseline characteristics of new users of antidepressants aged 75 and above (*N* = 185,225)

Characteristics	*N* (%)
Women	117,606 (63.5)
Mean age, median (age range)	83.4, 83 (75–110)
Age group	
75–79	53,126 (28.7)
80–84	56,223 (30.4)
85–89	47,678 (25.7)
≥ 90	28,198 (15.2)
Resident in nursing home	34,268 (18.5)
Previous suicide attempt	763 (0.4)
Diagnosis of depression in specialised care	7902 (4.3)
Received multidose dispensed medications	96,049 (51.9)
Type of antidepressant	
SSRI	116,870 (63.1)
Citalopram	90,427 (48.8)
Sertraline	18,373 (9.9)
Escitalopram	6150 (3.3)
SNRI/NRI	46,883 (25.3)
Mirtazapine	41,294 (22.3)
Duloxetine	2319 (1.3)
TCA	21,440 (11.6)
Amitriptyline	20,651 (11.1)
Other	32 (0.02)
Concomitant use of psychotropic medications	105,114 (56.8)
Anxiolytics	93,795 (50.6)
Hypnotics	68,180 (36.8)
Anti-dementia drugs	12,766 (6.9)
Antipsychotics	12,608 (6.8)
Mood stabilisers	5420 (2.9)
Use of statins	52,358 (28.3)

*TCA* tricyclic antidepressant, *SSRI* selective serotonin reuptake inhibitor, *SNRI*/*NRI* serotonin-noradrenaline reuptake inhibitor/Norepinephrine reuptake inhibitor

Overall, 90,681 persons (48.7%) died during follow-up. Suicide was the cause of death for 295 individuals (199 men and 96 women), corresponding to an incidence rate of 50 per 100,000 person-years for the total study population (106 per 100,000 person-years in men, and 25 per 100,000 person-years in women). Suicide methods are presented for men and women in Online Resource 3.

Table 2 shows that the adjusted SHR (aSHR) for suicide was doubled among individuals who switched to another antidepressant medication (aSHR 2.42, 95% confidence interval 1.65 to 3.55) compared to those who did not. Elevated aSHRs were observed in those who filled a concomitant prescription for anxiolytics (1.54, 1.20 to 1.96), hypnotics (2.20, 1.69 to 2.85), and antipsychotics (1.73, 1.19 to 2.51) compared to those with no fill for each group.

**Table 2:** Unadjusted and adjusted sub-hazard ratios for suicide by use patterns of antidepressants in a national cohort of new users aged 75 and above

Variable	*N* suicide	Unadjusted SHR (95% CI)	*P* value	Adjusted SHR (95% CI)^a^	*P* value
Early discontinuation^b^	12	0.74 (0.42–1.32)	0.31	0.82 (0.46–1.42)	0.50
Combination use of ≥ 2 antidepressants^c^	16	2.31 (1.39–3.84)	0.001	1.28 (0.74–2.22)	0.38
Switch to another antidepressant^d^	35	3.37 (2.35–4.82)	< 0.001	2.42 (1.65–3.55)	< 0.001
Medication possession ratio of antidepressants < 80%^e^	85	1.24 (0.97–1.58)	0.08	1.02 (0.79–1.10)	0.89
Concomitant use of psychotropic medications^f^	241				
Hypnotics	207	2.57 (1.99–3.30)	< 0.001	2.20 (1.69–2.85)	< 0.001
Anxiolytics	154	2.18 (1.72–2.77)	< 0.001	1.54 (1.20–1.96)	< 0.001
Antipsychotics	39	1.38 (1.98–1.93)	0.06	1.73 (1.19–2.51)	0.004
Anti-dementia drugs	14	0.43 (0.25–0.73)	0.002	0.72 (0.42–1.24)	0.24
Mood stabilisers	5	0.79 (0.32–1.89)	0.58	0.74 (0.30–1.79)	0.50

.Due to partially missing data, 210 persons were excluded from the regression analysis

*SHR* sub-hazard ratio

^a^Adjusted for age, sex, suicide attempt within 1 year preceding the index date, serious depression, use of statins (a proxy of cardiovascular comorbidity), and nursing home residence

^b^Reference group: those who did not discontinue their treatment within 180 days following the index date

^c^Reference group: those who did not combine two antidepressants within 180 days following the index date

^d^Reference group: those who did not switch to another antidepressant within 180 days following the index date

^e^The proportion of days covered by antidepressant medications during the follow-up period. (Threshold of MPR to define adherence ≥ 80%)

^f^Reference group: those who did not use the specified psychotropic medication within 90 days following the refill of an antidepressant

There were 654 suicide attempts during the follow-up period (300 men and 354 women), yielding an incidence rate of 117 per 100,000 person-years for the total study population (167 per 100,000 person-years in men and 94 per 100,000 person-years in women).

Table 3 shows that the risk of suicide attempt was almost doubled among those who switched to another antidepressant (1.76, 1.32 to 2.34). Elevated aSHRs were observed for those who concomitantly filled a prescription for anxiolytics (2.04, 1.73 to 2.40) and hypnotics (2.86, 2.38 to 3.43). The aSHR for suicide attempt was significantly lower among individuals who were prescribed anti-dementia drugs (0.40, 0.27 to 0.59) compared to those without such drugs. There was no significant association between refill adherence (MPR ≥ 80%) and suicidal behaviours in both adjusted and unadjusted analyses.

**Table 3:** Unadjusted and adjusted sub-hazard ratios for suicide attempts by use patterns of antidepressants in a national cohort of new users aged 75 and above

Variable	*N* suicide attempts	Unadjusted SHR (95% CI)	*P* value	Adjusted SHR (95% CI)^a^	*P* value
Early discontinuation^b^	29	0.81 (0.47–1.18)	0.27	1.14 (0.78–1.66)	0.50
Combination use of ≥ 2 antidepressants^c^	27	1.71 (1.17–2.54)	0.01	0.91 (0.60–1.37)	0.65
Switch to another antidepressant^d^	58	2.40 (1.82–3.15)	< 0.001	1.76 (1.32–2.34)	< 0.001
Medication possession ratio of antidepressants ≥ 80%^e^	432	0.99 (0.84–1.15)	0.85	0.87 (0.73–1.03)	0.10
Concomitant use of psychotropic medications^f^	578				
Hypnotics	500	3.35 (2.96–4.23)	< 0.001	2.86 (2.38–3.43)	< 0.001
Anxiolytics	396	2.42 (2.06–2.85)	< 0.001	2.04 (1.73–2.40)	< 0.001
Antipsychotics	96	2.37 (2.17–2.60)	< 0.001	1.16 (0.91–1.48)	0.24
Anti-dementia drugs	27	0.36 (0.25–0.54)	< 0.001	0.40 (0.27–0.59)	< 0.001
Mood stabilisers	21	1.49 (2.73–3.12)	0.07	1.26 (0.81–1.95)	0.31

Due to partially missing data, 210 persons were excluded from the regression analysis

*SHR* sub-hazard ratio

^a^Adjusted for age, sex, suicide attempt within 1 year preceding the index date, serious depression, use of statins (a proxy of cardiovascular comorbidity), and nursing home residence

^b^Reference group: those who did not discontinue their treatment within 180 days following the index date

^c^Reference group: those who did not combine two antidepressants within 180 days following the index date

^d^Reference group: those who did not switch to another antidepressant within 180 days following the index date

^e^The proportion of days covered by antidepressant medications during the follow-up period. (Threshold of MPR to define adherence ≥ 80%)

^f^Reference group: those who did not use the specified psychotropic medication within 90 days following the refill of an antidepressant

Similar results were obtained for the associations between the use patterns of antidepressants and suicide in the gender-stratified analyses (Online Resource 4). However, a decreased risk of suicide in those using anti-dementia drugs was observed in men only. For the outcome suicide attempt, an increased risk was found among women using mood stabilisers but not in men. Several associations were no longer significant, which could be related to the relatively small number of cases in the stratified analyses.

The results from the Cox regression were in accordance with the Fine and Gray regression and are presented in the Online Resource 5.

## Discussion

In this national population-based cohort study of all new users of antidepressants aged 75 years and older, we found increased risk of both fatal and non-fatal suicidal behaviour among those who concomitantly used anxiolytics and hypnotics and among those who switched antidepressant medication within the first 6 months of treatment.

The study is based on national data with no exclusion criteria, minimising the risk of selection bias. Moreover, there is no recall bias as use patterns and potential confounders were register-based. While we adjusted our regression models for several known confounders, our study design did not allow investigating other factors, such as substance misuse and family history of psychiatric disorder or suicidal behaviour. Nor did we have details about the psychopathology that may have alerted physicians to the need for treatment at index. Therefore, indication biases are to be considered [28]. The decision to prescribe a particular antidepressant or other psychotropic medication, and at a certain dose to a specific patient, depends upon the clinical diagnosis, or the severity of depression and concomitant medical conditions, which may be itself associated with the occurrence of the suicidal behaviours.

We used the term serious depression to describe the cases identified through the National Patient Register since the common practice in Sweden is that milder forms of depression are treated within primary care and more serious cases are referred [29]. However, some serious depressions may be managed in primary care only and are therefore missed in the National Patient Register. Also, milder forms of depression are associated with suicidal behaviour in older adults [30, 31], and these cannot be captured with our study design as diagnostic data from primary care were lacking in national registers, nor can we distinguish between new users who initiated antidepressant treatment for the very first time and previous users who were off antidepressants during the year prior to index. Another consideration is that we could not account for variation in depression severity during follow-up. We must therefore consider the possibility of residual confounding. The absence of association between refill adherence and suicidal behaviour should be interpreted with caution. Non-users of antidepressants may have been classified as users if they filled a prescription but failed to ingest the medication. We should highlight that half of the study population used multidose dispensed medications (automatic refill) which creates an artificially regular drug-dispensing pattern and thereby a high refill adherence. The small number of suicides and suicide attempts made it unfeasible to analyse results for individual substances. We did not also account for the period of increased risks to suicidal behaviours following the switch to another antidepressant, or for possible time trends in the associations between use pattern and suicidal behaviours. Further, we could not conduct stratified analyses by age groups, which is a limitation as factors related to suicidal behaviour in extreme old age may differ from those for persons in their mid-70s and 80s [32]. Our gender-stratified analyses should also be interpreted with caution due to the low number of suicides in women and therefore low power. By restricting the study to new users, in line with guidelines such as the GRACE guidelines [33], confounding can be limited yet it may limit the generalisability of our findings to all users of antidepressants. Excluding prevalent users may not allow evaluation of risks related to chronic exposure to antidepressants. However, the relatively long follow-up period means that some persons can be considered long-term users.

Results from this large prospective study expand on our previous findings pointing to increased risk of suicidal behaviour in older adults who used sedatives and hypnotics [15]. One possible explanation for the observed risk increase may be that these drugs trigger an aggressive behaviour [34]. It is also possible that concomitant use of anxiolytics and hypnotics is merely a marker for some other factors related to suicide risk or self-harm, for example, more complex psychopathology [35, 36]. In previous population-based studies in which older adults took part in psychiatric examinations, we have shown that anxiety symptoms [37], as well as sleep problems [32], may be independent predictors of suicidality. Furthermore, as older adults of today are less likely to abstain from alcohol compared to earlier born cohorts [38], possible interactions between these medications and alcohol cannot be outruled. Alcohol use may intensify impulsive tendencies, thereby increasing risk of suicidal behaviour.

Our finding of elevated SHRs of suicide and suicide attempt associated with switching antidepressants may in part be explained by the fact that drug cessation can in some cases result in withdrawal symptoms and relapse or exacerbation of the depression [39]. We cannot rule out confounding by indication, and switching may in many cases be perceived as a marker of treatment-resistant depression [40]. Moreover, patients with severe depression may transiently become agitated and restless and harm themselves (with or without fatal outcome), before the new antidepressant starts to relieve depression.

Confounding by indication may also help to explain the higher risk of suicide among those taking antipsychotics, as older adults with psychotic depression may be at particular risk of suicidal behaviour. Another factor to be taken into account is that the elevated risk could be in part related to akathisia [41, 42]. Older patients are more prone to drug-induced movement disorders, but they may lack the vocabulary to describe this adverse event. The lower rates of suicide attempts among new users of antidepressants concomitantly taking anti-dementia drugs is also likely to be explained, at least in part, by confounding by indication. Antidepressants are often prescribed well into the course of dementia also for other conditions such as psychomotor agitation. Clinical studies indicate that cognition may be compromised in non-demented older adults with suicidal behaviour [43], and it is possible that anti-dementia treatment might have a preventive effect for suicidal behaviour for some individuals facing issues of milder cognitive impairment. One Danish cohort study that included also younger patients demonstrated increased risk for suicide when a first diagnosis of dementia was made in hospital [44]. However, the patients in that study most likely represented a high-risk subgroup. A previous population register-based study reported a lower risk of suicide among women who discontinued their antidepressant treatment, but the age range of the study population was broad, including persons aged 50 and above [45]. As prescribing patterns are different among older age groups with lower life expectancy [46], it may not be relevant to compare the findings of our study with those conducted among younger adult populations.

The rate of suicide in our population is more than twofold that observed in the general population aged 75 years and above [47]. Our findings may help to inform the prescribers who initiate antidepressant treatment in their older adult patients. Inconsistent results regarding the associations between the use of antipsychotics and fatal and non-fatal suicidal behaviours indicate a need for further research to draw more definite conclusions about these associations. The same applies to the findings regarding anti-dementia drugs. Future research should consider indication biases and the clinical characteristics of patients initiating antidepressant therapy. The latter includes severity of depression symptoms as well as anxiety, cognitive dysfunction, and overall frailty. Studies need to be carried out in other settings as availability of healthcare, prescription patterns, and rates of both fatal and non-fatal suicidal behaviour vary widely in a global perspective.

### Contribution of authors

KH conceived and designed the study, helped acquire the data, helped with data analysis and interpretation of results, drafted the article, and had the final approval. KAS conceived and designed the study, helped acquire the data, helped with data analysis, revised the article for content, and gave the final approval for publication. AH helped with data analysis and gave the final approval for publication. IS helped with interpretation of results, revised the manuscript, and gave the final approval for publication. SG performed data analysis, helped with interpretation of results, and gave the final approval for publication. MW is the primary investigator of the project. She conceived and designed the study, helped acquire the data, helped with interpretation of results, revised the article for content, and gave the final approval for publication. All authors had full access to all of the data (including statistical reports and tables) and can take responsibility for the integrity of the data and the accuracy of the data analysis. KH is the guarantor.

## Electronic supplementary material

ESM 1 (PDF 452 kb)

ESM 2 (PDF 221 kb)

ESM 3 (PDF 154 kb)

ESM 4 (PDF 361 kb)

ESM 5 (PDF 214 kb)
