# Supplementary figures and images for: Antidepressants and suicidal behaviour in late life: a prospective population-based study of use patterns in new users aged 75 and above
Source: Eur J Clin Pharmacol. 2017 Nov 4;74(2):201–8. doi: 10.1007/s00228-017-2360-x (PMC5765190; doi:10.1007/s00228-017-2360-x)

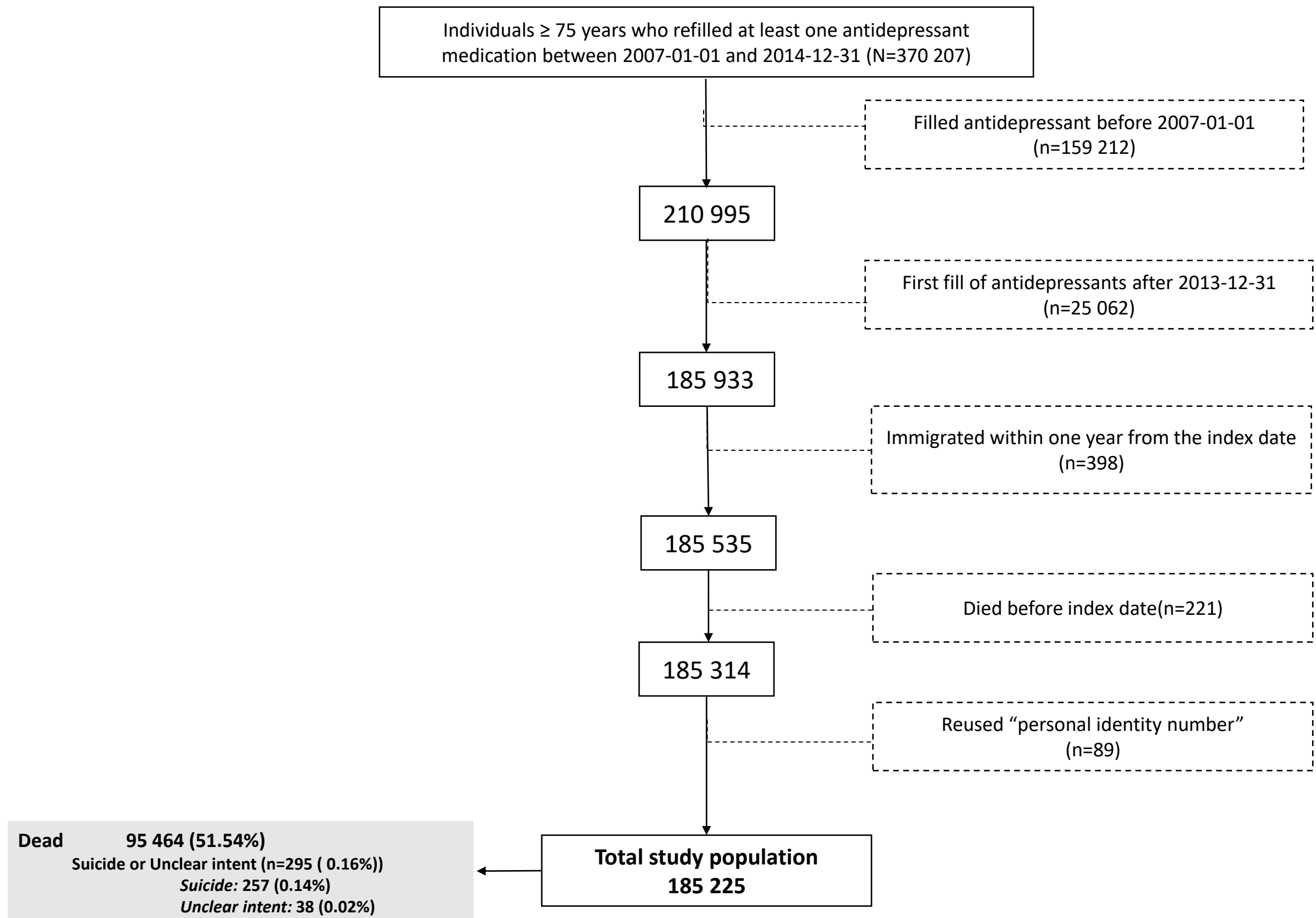

Online Resource 1. Study flow diagram

Supplement: Supplementary file 1 — (PDF 452 kb) [file 228_2017_2360_MOESM1_ESM.pdf]
