# Supplementary material for: Antidepressants and suicidal behaviour in late life: a prospective population-based study of use patterns in new users aged 75 and above
Source: Eur J Clin Pharmacol. 2017 Nov 4;74(2):201–8. doi: 10.1007/s00228-017-2360-x (PMC5765190; doi:10.1007/s00228-017-2360-x)
Supplement: Supplementary file 2 — (PDF 221 kb) [file 228_2017_2360_MOESM2_ESM.pdf]

Online Resource 2

Table 1. Crude associations between use patterns of antidepressants and baseline characteristics including index medication

		Early discontinuation		Combination use		Switch to another antidepressant		Concomitant use of psychotropic medications		Antipsychotics		Hypnotics		Anti-dementia drugs		Mood stabilisers		Anxiolytics		Total
		N	%[a]	N	%[a]	N	%[a]	N	%[a]	N	%[a]	N	%[a]	N	%[a]	N	%[a]	N	%[a]	N
**Antidepressant medication at index date**	**TCA**	2094	9,8%	299	1,4%	554	2,6%	14445	67,4%	1512	7,1%	11508	53,7%	997	4,7%	630	2,9%	7696	35,9%	21440
	**SSRI**	4212	3,6%	3760	3,2%	6199	5,3%	92465	79,1%	19820	17,0%	63563	54,4%	18159	15,5%	3574	3,1%	62121	53,2%	116870
	**SNRI / NRI**	1706	3,6%	1749	3,7%	2092	4,5%	38094	81,3%	8588	18,3%	28378	60,5%	6297	13,4%	1255	2,7%	24967	53,3%	46883
	**Other**	4	12,5%	0	0,0%	2	6,3%	29	90,6%	3	9,4%	23	71,9%	7	21,9%	3	9,4%	21	65,6%	32
**Nursing home residence**	**No**	7885	4,8%	5051	3,1%	7943	4,9%	127608	78,1%	24301	14,9%	92768	56,8%	21641	13,2%	4753	2,9%	82711	50,6%	163381
	**Yes**	131	0,6%	757	3,5%	904	4,1%	17425	79,8%	5622	25,7%	10704	49,0%	3819	17,5%	709	3,2%	12094	55,4%	21844
**Sex**	**Male**	2776	4,1%	2220	3,3%	3072	4,5%	50963	75,4%	10948	16,2%	36674	54,2%	9210	13,6%	2374	3,5%	31745	46,9%	67619
	**Female**	5240	4,5%	3588	3,1%	5775	4,9%	94070	80,0%	18975	16,1%	66798	56,8%	16250	13,8%	3088	2,6%	63060	53,6%	117606
**Age group**	**75-79**	3176	6,0%	1627	3,1%	2743	5,2%	40910	77,0%	6920	13,0%	29609	55,7%	7476	14,1%	1944	3,7%	26130	49,2%	53126
	**80-84**	2619	4,7%	1799	3,2%	2815	5,0%	44473	79,1%	9100	16,2%	31744	56,5%	9096	16,2%	1781	3,2%	28853	51,3%	56223
	**85-89**	1606	3,4%	1541	3,2%	2134	4,5%	37601	78,9%	8578	18,0%	26641	55,9%	6653	14,0%	1241	2,6%	24814	52,0%	47678
	**≥90**	615	2,2%	841	3,0%	1155	4,1%	22049	78,2%	5325	18,9%	15478	54,9%	2235	7,9%	496	1,8%	15008	53,2%	28198

TCA: Tricyclic antidepressant; SSRI: Selective serotonin reuptake inhibitor; SNRI/NRI: Serotonin–noradrenaline reuptake inhibitor/Norepinephrine reuptake inhibitor.

[a] Percentage within each use pattern.
