# Supplementary material for: Antidepressants and suicidal behaviour in late life: a prospective population-based study of use patterns in new users aged 75 and above
Source: Eur J Clin Pharmacol. 2017 Nov 4;74(2):201–8. doi: 10.1007/s00228-017-2360-x (PMC5765190; doi:10.1007/s00228-017-2360-x)
Supplement: Supplementary file 3 — (PDF 154 kb) [file 228_2017_2360_MOESM3_ESM.pdf]

Online Resource 3. Table 1. Methods of suicide among the study population (N=295)*

**Method of suicide**	**Number of suicides (%)**	**Number of suicides among men (%)**	**Number of suicides among women (%)**
**Hanging, strangulation and suffocation**	87 (29)	76 (38)	11 (11)
**Poisoning**	78 (26)	36 (18)	42 (44)
**Jumping from a high place**	36 (12)	23 (12)	13 (14)
**Drowning and submersion**	35 (12)	15 (8)	20 (21)
**Firearm**	31 (11)	31 (16)	0 (0)
**Sharp object**	6 (2)	5 (3)	1 (1)
**Moving object**	5 (2)	4 (2)	1 (1)
**Other**	17 (6)	9 (5)	8 (8)
**Total**	295	199	96

*Based on the International classification of Diseases (ICD-10) from the Cause of Death Register. Both diagnoses of international self-harm and of undetermined intent were included.

Online Resource 3. Table 2. Methods of suicide attempt among the study population (N=654)*

Method of suicide attempt	Number of suicide attempts (%)	Number of suicide attempts among men (%)	Number of suicide attempts among women (%)
**Poisoning**	509 (78)	201 (67)	308 (87)
**Sharp object**	82 (13)	55 (18)	27 (8)
**Hanging, strangulation and suffocation**	19 (3)	16 (5)	3 (1)
**Jumping from a high place**	15 (2)	10 (3)	5 (1)
**Drowning and submersion**	10 (2)	6 (2)	4 (1)
**Moving object**	4 (1)	4 (1)	0 (0)
**Other**	15 (3)	8 (3)	7 (2)
**Total**	654	300	354

*Based on the International classification of Diseases (ICD-10) from the National Patient Register. Both diagnoses of international self-harm and of undetermined intent were included.
