# Supplementary material for: Antidepressants and suicidal behaviour in late life: a prospective population-based study of use patterns in new users aged 75 and above
Source: Eur J Clin Pharmacol. 2017 Nov 4;74(2):201–8. doi: 10.1007/s00228-017-2360-x (PMC5765190; doi:10.1007/s00228-017-2360-x)
Supplement: Supplementary file 4 — (PDF 361 kb) [file 228_2017_2360_MOESM4_ESM.pdf]

Online Resource 4. Table 1. Adjusted sub-hazard ratios for suicide by use patterns of antidepressants, stratified by gender

Variable	Men, Suicides = 199		Women, Suicides = 96	
	Adjusted SHR[a] (95% CI)	P	Adjusted SHR[a] (95% CI)[a]	P
**Early discontinuation**[b]	0.77 (0.36-1.66)	0.51	1.15 (0.46-2.84)	0.77
**Combination use of ≥ 2 antidepressants**[c]	0.96 (0.47-1.95)	0.90	1.84 (0.78-3.38)	0.17
**Switch to another antidepressant**[d]	3.03 (1.93-4.76)	<0.001	1.76 (0.88-3.53)	0.11
**Medication possession ratio of antidepressants ≥80%**[e]	0.91 (0.67-1.22)	0.60	1.33 (0.84-2.12)	0.23
**Concomitant use of psychotropic medications**[f]				
**Hypnotics**	2.13 (1.56-2.93)	<0.001	2.46 (1.57-3.88)	0.001
**Anxiolytics**	1.68 (1.24-2.27)	<0.001	1.15 (0.76-1.76)	0.51
**Antipsychotics**	0.87 (0.54-1.39)	0.55	1.86 (1.07-3.20)	0.03
**Anti-dementia drugs**	0.37 (0.18-0.76)	0.01	0.55 (0.23-1.30)	0.17
**Mood stabilisers**[g]	0.90 (0.37-2.20)	0.68	-	-

SHR: Sub-hazard ratio

[a] Adjusted for age, sex, suicide attempt within one year preceding the index date, serious depression, use of statins (a proxy of cardiovascular comorbidity), and nursing home residence.

[b] Reference group: those who did not discontinue their treatment within 180 days following the index date

[c] Reference group: those who did not combine two antidepressants within 180 days following the index date

[d] Reference group: those who did not switch to another antidepressant within 180 days following the index date

[e] The proportion of days covered by antidepressant medications during the follow-up period. (Threshold of MPR to define adherence ≥ 80%)

[f] Reference group: those who did not use the specified psychotropic medication within 90 days following the refill of an antidepressant

[g] The low number of suicides did not allow to conduct the regression analysis among women

Online Resource 4. Table 2. Adjusted sub-hazard ratios for suicide attempts by use patterns of antidepressants, stratified by gender

Variable	Men, attempts = 300		Women, attempts = 354	
	Adjusted SHR[a] (95% CI)	P	Adjusted SHR[a] (95% CI)	P
**Early discontinuation**[b]	1.00 (0.56-1.79)	0.1	1.18 (0.71-1.94)	0.52
**Combination use of ≥ 2 antidepressants**[c]	1.27 (0.74-2.17)	0.38	0.63 (0.33-1.22)	0.17
**Switch to another antidepressant**[d]	1.40 (0.87-2.26)	0.16	1.97 (1.38-2.82)	<0.001
**Medication possession ratio of antidepressants ≥80%**[e]	0.82 (0.64-1.05)	0.12	0.90 (0.72-1.13)	0.38
**Concomitant use of psychotropic medications**[f]				
**Hypnotics**	2.86 (2.19-3.72)	<0.001	2.83 (2.20-3.65)	<0.001
**Anxiolytics**	1.88 (1.48-2.37)	<0.001	2.25 (1.78-2.85)	<0.001
**Antipsychotics**	1.37 (0.98-1.93)	0.07	1.16 (0.83-1.62)	0.38
**Anti-dementia drugs**	0.49 (0.29-0.84)	0.01	0.34 (0.19-0.62)	<0.001
**Mood stabilisers**	0.68 (0.30-1.55)	0.36	1.99 (1.18-3.37)	0.01

SHR: Sub-hazard ratio

[a] Adjusted for age, sex, suicide attempt within one year preceding the index date, serious depression, use of statins (a proxy of cardiovascular comorbidity), and nursing home residence.

[b] Reference group: those who did not discontinue their treatment within 180 days following the index date

[c] Reference group: those who did not combine two antidepressants within 180 days following the index date

[d] Reference group: those who did not switch to another antidepressant within 180 days following the index date

[e] The proportion of days covered by antidepressant medications during the follow-up period. (Threshold of MPR to define adherence ≥ 80%)

[f] Reference group: those who did not use the specified psychotropic medication within 90 days following the refill of an antidepressant
