# Supplementary material for: Antidepressants and suicidal behaviour in late life: a prospective population-based study of use patterns in new users aged 75 and above
Source: Eur J Clin Pharmacol. 2017 Nov 4;74(2):201–8. doi: 10.1007/s00228-017-2360-x (PMC5765190; doi:10.1007/s00228-017-2360-x)
Supplement: Supplementary file 5 — (PDF 214 kb) [file 228_2017_2360_MOESM5_ESM.pdf]

Online Resource 5. Table 1. Cox hazard ratios for suicide and suicide attempts by use patterns of antidepressants*

Variable	Suicide=294		Suicide attempts=654	
	**Unadjusted HR (95% CI)**	**Adjusted HR[a] (95% CI)**	**Undjusted HR (95% CI)**	**Adjusted HR[a] (95% CI)**
**Early discontinuation[b]**	0.71 (0.40-1.26)	0.86 (0.48-1.55)	0.77 (0.53-1.11)	1.07 (0.73-1.56)
**Combination use of ≥ 2 antidepressants[c]**	2.36 (1.42-3.91)	1.21 (0.71-2.06)	1.76 (1.20-2.60)	0.90 (0.60-1.35)
**Switch to another antidepressant[d]**	3.36 (2.35-4.80)	2.49 (1.71-3.62)	2.39 (1.82-3.14)	1.71 (1.29-2.27)
**Medication possession ratio of antidepressants ≥80%[e]**	1.25 (0.97-1.62)	1.05 (0.81-1.37)	0.99 (0.84-1.17)	0.86 (0.73-1.02)
**Concomitant use of psychotropic medications[f]**				
**Hypnotics**	2.57 (1.99-3.31)	2.21 (1.71-2.87)	3.53 (2.94-4.25)	2.81 (2.33-3.39)
**Anxiolytics**	1.75 (1.38-2.21)	1.51 (1.18-1.92)	2.47 (2.11-2.90)	2.08 (1.76-2.45)
**Antipsychotics**	1.51 (1.07-2.11)	1.24 (0.87-1.76)	1.67 (1.36-2.11)	1.28 (1.02-1.60)
**Anti-dementia drugs**	0.43 (0.25-0.74)	0.43 (0.25-0.74)	0.37 (0.25-0.55)	0.40 (0.27-0.59)
**Mood stabilisers**	0.80 (0.33-1.95)	0.66 (0.27-1.60)	1.54 (1.00-2.38)	1.30 (0.84-2.02)

SHR: Sub-hazard ratio

*Due to partially missing data, 210 persons were excluded from the regression analysis

[a] Adjusted for age, sex, suicide attempt within one year preceding the index date, serious depression, use of statins (a proxy of cardiovascular comorbidity), and nursing home residence

[b] Reference group: those who did not discontinue their treatment within 180 days following the index date

[c] Reference group: those who did not combine two antidepressants within 180 days following the index date

[d] Reference group: those who did not switch to another antidepressant within 180 days following the index date

[e] The proportion of days covered by antidepressant medications during the follow-up period. (Threshold of MPR to define adherence ≥ 80%)

[f] Reference group: those who did not use the specified psychotropic medication within 90 days following the refill of an antidepressant
